# Individual participant data meta-analyses should not ignore clustering

**DOI:** 10.1016/j.jclinepi.2012.12.017

**Published:** 2013-08

**Authors:** Ghada Abo-Zaid, Boliang Guo, Jonathan J. Deeks, Thomas P.A. Debray, Ewout W. Steyerberg, Karel G.M. Moons, Richard David Riley

**Affiliations:** a European Centre for Environment and Human Health, Peninsula College of Medicine and Dentistry, University of Exeter, Knowledge Spa, Royal Cornwall Hospital, Truro, Cornwall TR1 3HD, UK; b Faculty of Medicine and Health Sciences, School of Community Health Sciences, The University of Nottingham, Sir Colin Campbell Building, Jubilee Campus, Wollaton Road, Nottingham NG8 1BB, UK; c Public Health, Epidemiology & Biostatistics, School of Health and Population Sciences, The Public Health Building, University of Birmingham, Birmingham B15 2TT, UK; d Julius Center for Health Sciences and Primary Care, University Medical Center Utrecht, Utrecht, The Netherlands; e Department of Public Health, Erasmus MC, PO Box 2040, 3000 CA Rotterdam, The Netherlands

**Keywords:** Individual participant data meta-analysis, Individual patient data, Evidence synthesis, Cluster, Simulation, Binary outcome, Pooled analysis

## Abstract

**Objectives:**

Individual participant data (IPD) meta-analyses often analyze their IPD as if coming from a single study. We compare this approach with analyses that rather account for clustering of patients within studies.

**Study Design and Setting:**

Comparison of effect estimates from logistic regression models in real and simulated examples.

**Results:**

The estimated prognostic effect of age in patients with traumatic brain injury is similar, regardless of whether clustering is accounted for. However, a family history of thrombophilia is found to be a diagnostic marker of deep vein thrombosis [odds ratio, 1.30; 95% confidence interval (CI): 1.00, 1.70; *P* = 0.05] when clustering is accounted for but not when it is ignored (odds ratio, 1.06; 95% CI: 0.83, 1.37; *P* = 0.64). Similarly, the treatment effect of nicotine gum on smoking cessation is severely attenuated when clustering is ignored (odds ratio, 1.40; 95% CI: 1.02, 1.92) rather than accounted for (odds ratio, 1.80; 95% CI: 1.29, 2.52). Simulations show models accounting for clustering perform consistently well, but downwardly biased effect estimates and low coverage can occur when ignoring clustering.

**Conclusion:**

Researchers must routinely account for clustering in IPD meta-analyses; otherwise, misleading effect estimates and conclusions may arise.

**What is new?**

**Key findings**
• When meta-analyzing individual participant data (IPD) from multiple studies, our findings show that statistical and clinical conclusions can change depending on whether the analysis accounts for the clustering of patients within studies. When synthesizing IPD from observational studies in deep vein thrombosis (DVT), a meta-analysis ignoring clustering leads to a potentially important diagnostic marker for DVT being missed. When synthesizing IPD from randomized trials of treatment for smoking cessation, the effect of nicotine gum on smoking cessation is severely underestimated when clustering is ignored.

**What this adds to what was known?**
• It is inappropriate to simply ignore the clustering of patients within studies and analyze the IPD as if coming from a single study. When there is large variability in baseline risk, logistic regression simulations show that this naive approach leads to a downward bias in effect estimates, with small standard errors that produce a low coverage substantially less than 95%; this problem becomes worse as the true effect size increases. Other mechanisms may also cause analyses ignoring clustering to perform poorly, such as between-study heterogeneity in effect or covariate patterns. In contrast, one-step or two-step IPD meta-analyses that account for clustering generally perform consistently well.

**What is the implication, and what should change now?**
• Researchers synthesizing IPD from multiple studies should account for the clustering of patients within different studies; otherwise, misleading effects estimates and coverage and potentially inappropriate clinical conclusions may arise.

## 1 Introduction

Individual participant data (IPD) meta-analysis refers to when participant-level data are obtained from multiple studies and then synthesized [1]. This contrasts the usual meta-analysis approach, which obtains and then synthesizes aggregate data (such as a treatment effect estimates) extracted from study publication or study authors [2]. IPD offers many potential advantages for the meta-analyst [1–3]; in particular, it reduces reliance on the reporting quality of individual studies as, with the raw data at hand, the meta-analyst can be more flexible and consistent in their choice of analysis method, can estimate directly the effect estimates of interest, and better account for study heterogeneity and subgroup effects.

Methods for IPD meta-analysis use either a one-step or a two-step approach [4]. In the two-step approach, the IPD are first analyzed separately in each study using an appropriate statistical method for the type of data being analyzed. For example, to assess the association between a continuous factor (e.g., age) and the odds of a binary outcome (e.g., death), a logistic regression model might be fitted, to produce aggregate data for each study, such as the odds ratio and its associated standard error; these are then synthesized in the second step using a suitable model for meta-analysis of aggregate data, such as one weighting by the inverse of the variance while assuming fixed or random effects across studies. In the one-step approach, the IPD from all studies are modeled simultaneously; this again requires a model specific to the type of data being synthesized, alongside appropriate specification of the meta-analysis assumptions (e.g., fixed or random effects across studies). Clustering of patients within studies can be accounted for by stratifying the analysis by study (i.e., by estimating a separate intercept for each study) or assuming that the study intercepts (baseline risk) are randomly drawn from some distribution.

Many existing articles discuss the implementation and merits of one-step and two-step IPD meta-analysis methods [5–11], and the methods often give very similar results [10,12,13]. For example, for time-to-event data, Tudur Smith and Williamson [14] show through simulation that when there is no heterogeneity in effect and the proportional hazards assumption holds, a one-step stratified Cox model produces similar effect estimates to the two-step (inverse variance weighted) approach. For continuous outcome data analyzed using linear models, Olkin and Sampson [12] and subsequently Matthew and Nordstrom [13,15] show that the one-step and two-step approaches provide identical results when estimating a treatment effect under certain theoretical conditions; although when covariates are added, differences may occur. Jones et al. [9] consider longitudinal continuous outcome data and empirically show that the one-step and two-step approaches produce similar effect estimates, as long as correlations between time points are incorporated. For binary outcome data, there may be some advantage of a one-step approach when the event risk or rate is low or the sample size is small; in contrast to the two-step approach, the one-step approach allows the exact binomial distribution to be used and does not require continuity corrections when zero events occur [16,17].

However, potentially of more concern than the choice of one-step or two-step approach, is that there is growing evidence that researchers undertake the one-step approach but ignore the clustering of patients within studies, thereby treating the IPD as if it all came from one study. For example, Simmonds et al. [4] examined IPD meta-analyses of randomized trials and found that 3 of 14 using a one-step approach ignored clustering. Similarly, Abo-Zaid et al. [18] examined IPD meta-analyses of prognostic factor studies and found that 5 of 11 using a one-step approach did not state that they accounted for clustering.

Using real examples and through simulation, we therefore studied the potential impact of ignoring clustering on IPD meta-analysis results and report our findings in this article. We focus on IPD meta-analyses aimed at quantifying whether a single (continuous or binary) factor or determinant of interest is associated with (the odds of) a binary outcome. For example, one may wish to summarize the outcome risk in a treatment group relative to the control group (i.e., estimate a treatment effect); estimate whether a certain prognostic marker is associated with future event risk (i.e., estimate a prognostic effect); or quantify whether the presence of a certain diagnostic test result increases or decreases the probability of having a particular disease. These are common situations in the (IPD) meta-analysis field. In Section 2, we introduce three one-step and two-step models of interest, and in Section 3, we apply them to three real applications. The performance of the one-step methods is evaluated through simulation in Section 4, and we then conclude with Discussion and recommendations.

## 2 One-step and two-step IPD meta-analysis approaches

Consider that there are *i* = 1 to *m* independent studies that each assess the binary outcome of interest for *n*_*i*_ participants. Let *y*_*ik*_ be the outcome (1, event; 0, no event) of participant *k* in study *i*, where *k* = 1 to *n*_*i*_, and let *x*_*ik*_ be a participant-level factor (covariate), which could be continuous or binary. We term an “IPD study” one that provides *y*_*ik*_ and *x*_*ik*_ for the *n*_*i*_ participants in the study. Note that, for a binary factor, if the number of participants and events for each of the two categories are known, then IPD for these two variables can simply be reconstructed by creating a row for each participant and delegating them event responses and covariate status that collectively mirror the observed frequencies.

Given such IPD, there are a number of ways that researchers could estimate the summary risk or odds ratio across studies. We focus here on the use of a logistic regression framework, via a one-step approach ignoring clustering, a one-step approach accounting for clustering, or a two-step approach, as now described.

### 2.1 Model (1): one-step ignoring clustering

With this method, the IPD from all studies are stacked and analyzed together as if they were a single study; thus, the clustering of patients within different studies is ignored. The standard logistic model can be written as follows:

(1) $$\begin{array}{c} y_{ik}∼\text{Bernoulli}\left(p_{ik}\right)\\ \text{logit}\left(p_{ik}\right)=\alpha +\beta x_{ik}. \end{array}$$

The common *α* term for all studies shows that clustering is being ignored, and *α* can be interpreted as the log odds of the event for patients with *x*_*ik*_ equal to zero. The term *β* provides the log odds ratio comparing the odds of the event for two patients who differ in *x*_*ik*_ by one unit. Note that *β* is also assumed common to all studies, and so we have a fixed-effect meta-analysis here. We consider a random-effects approach and multivariable model extensions in our Discussion.

### 2.2 Model (2): one-step accounting for clustering

Here, the IPD from all studies are also stacked and analyzed together, but the clustering of patients within different studies is accounted for. The logistic model can be written as follows:

(2) $$\begin{array}{c} y_{ik}∼\text{Bernoulli}\left(p_{ik}\right)\\ \text{logit}\left(p_{ik}\right)=\alpha_{i}+\beta x_{ik}. \end{array}$$

Now the intercept term is not fixed, and *α*_*i*_ gives the log odds of the event in study *i* for those participants with *x*_*ik*_ equal to zero. The separate *α*_*i*_ term for each study shows that clustering per study is being accounted for at the baseline level, that is, each study is allowed to have their own baseline risk.

### 2.3 Model (3): two-step approach

Here, the IPD of each study is analyzed separately, and the log odds ratio estimates from each study are then combined (averaged) in an inverse variance–weighted fixed-effect meta-analysis, as follows:

(3) $$\begin{array}{l} \text{STEP}\;1\left(\text{each}\;\text{study}\;\text{separately}\right):\begin{array}{c} y_{ik}∼\text{Bernoulli}\left(p_{ik}\right)\\ \text{logit}\left(p_{ik}\right)=\alpha_{i}+\beta_{i}x_{ik}, \end{array}\\ \text{STEP}\;2\left(\text{meta-analysis}\;\text{of}\;\text{aggregatedata,}\;{\hat{\beta }}_{i}\text{s}\right):\begin{array}{c} {\hat{\beta }}_{i}=\beta +\varepsilon_{i}\\ \varepsilon_{i}∼N\left(0,\text{var}\left({\hat{\beta }}_{i}\right)\right). \end{array} \end{array}$$

By first analyzing each study separately, this approach automatically accounts for the clustering of patients within studies. In the second step, the var(${\hat{\beta }}_{i}$) estimates are assumed known, which is a common assumption in the meta-analysis field [19], and the pooled prognostic effect estimate ($\hat{\beta }$) will be a weighted average of the ${\hat{\beta }}_{i}$s, with study weights equal to the inverse of var(${\hat{\beta }}_{i}$) [20].

The parameters in equations (1) and (2), and those in both steps of equation (3), can be estimated using maximum likelihood (StataCorp, LP, College Station, TX, USA) [21]. Note that, when *x*_*ik*_ is a binary factor and the event risk is low and/or the sample size is small, some studies may have zero events for one of the factor's groups. The one-step approach accommodates such studies automatically through their contribution to the likelihood. However, the two-step approach first requires a so-called continuity correction (e.g., 0.5) to be added to all cells in such studies, to estimate a sensible log odds ratios and its standard error. This is a clear limitation of the two-step method, and this issue has been well discussed in the literature [22] and is not the focus of this article. We only consider examples without zero cells in this article.

## 3 Empirical IPD meta-analysis examples

We now introduce three motivating IPD meta-analysis examples to illustrate the potential similarities and differences of the models in meta-analyses of diagnostic studies, prognostic studies, and (randomized) therapeutic trials.

### 3.1 Mortality after traumatic brain injury

Hukkelhoven et al. [23] performed a meta-analysis of 14 prospective studies to assess the 6-month mortality risk in patients with traumatic brain injury (TBI). Their key objective was to examine the association between age and 6-month mortality risk. Biologically, this relationship is plausible as the adult brain is hypothesized to have decreased capacity for repair as it ages [24] because of a decreasing number of functioning neurons and a greater exposure to minor repetitive insults to the brain as age increases. In their meta-analysis, IPD were available for four studies (totaling 2,659 patients), containing the 6-month mortality outcome (dead or alive) and age for each patient in each study. These IPD are summarized in our Appendix A at www.jclinepi.com.

Of interest is the odds ratio comparing the odds of death by 6 months for two patients aged 10 years apart. Only a linear relationship with age was assumed. The results for each of models (1)–(3) are shown in Table 1, and there are only small unimportant statistical and clinical differences between them. Age is identified to have a statistically significant (*P* < 0.001) association with the odds of 6-month mortality in all models, and the odds ratio is 1.41 in the one-step model ignoring clustering and a slightly lower 1.37 in the two-step approach and one-step accounting for clustering. The standard error of the log odds ratio estimate is almost identical, 0.030 in the two-step and 0.029 in the others. There was no evidence of between-study heterogeneity in the odds ratio (*I*^2^ = 0), suggesting that the fixed-effect modeling assumption was appropriate. Based on this application alone, the observed findings might lead researchers to decide that it does not matter whether clustering is accounted for.

### 3.2 Diagnosis of deep vein thrombosis

IPD are available from six studies of patients with suspected deep vein thrombosis (DVT) [25–30] and of interest is whether a family history of thrombophilia (defined as yes or no) is associated with the risk of truly having DVT. One might expect patients with a family history of thrombophilia to be more likely to have a genuine DVT than those without. The studies are summarized in our Appendix A at www.jclinepi.com and contained a total of 4,599 patients of which 909 (19.8%) truly have DVT. The proportion of patients in each study with a family history of thrombophilia ranged from 0.03 to 0.26.

As in the TBI example, there is no heterogeneity (*I*^2^ = 0%), and the two-step and the one-step approaches accounting for clustering obtain similar estimates, standard errors, and confidence intervals (Table 2); they estimate that the odds of DVT are about 1.3 times higher for patients with a family history of thrombophilia, and the findings are (close to) statistically significant at the 5% level (*P* = 0.038 or 0.053). However, the one-step approach ignoring clustering estimates a much smaller odds ratio of 1.06, and there is now no statistically significant evidence that family history is an important risk factor (*P* = 0.64); the standard error of $\hat{\beta }$ is also smaller compared with that of the other models. Thus, in this example, the one-step approach ignoring clustering provides different statistical and clinical conclusions than the other approaches.

### 3.3 Smoking cessation and use of nicotine gum

Rice and Stead [31] perform a meta-analysis of 51 randomized trials to examine whether the use of nicotine gum increases the chances of stopping smoking. Altman and Deeks [32] used these trials to show the impact on the estimated number needed to treat when clustering of studies was ignored. We now extend this to consider the impact on the odds ratio. Specifically, for illustrative purposes, we consider a meta-analysis of just two of the trials (the same two used by Altman and Deeks), which are summarized in our Appendix A at www.jclinepi.com and the results shown in Table 3 (*I*^2^ = 14.3%). As in the DVT example, the one-step method ignoring clustering produces a smaller summary odds ratio (1.48) that is much closer to 1 than the other methods, which rather give estimates around 1.8 with wider confidence intervals.

## 4 Simulation methods

The above examples illustrate that the decision to account for clustering in IPD meta-analysis is potentially important. To look more generally at how ignoring clustering affects the statistical properties of estimates, we now present a simulation study of models (1) and (2).

### 4.1 Simulation procedure

Full details of our simulation are provided in our Appendix B at www.jclinepi.com. Briefly, for multiple scenarios, we simulated IPD (i.e., patient outcomes and prognostic factor values) for meta-analyses based on *m* = 5 or 10 studies; smaller (30–100 patients) or larger study sizes (up to 1,000 patients); a continuous or binary factor (*x*_*ik*_); a binary outcome *y*_*ik*_ (1, event; 0, alive), where *y*_*ik*_∼Benoulli(*p*_*ik*_) and $\text{logit}\left(p_{ik}\right)=\alpha_{i}+{\mathrm{\beta x}}_{ik}$; the chosen parameters of $\alpha_{i}∼N(\alpha ,\sigma_{\alpha }^{2})$; and for binary factors a *β* of 0, 0.1, or 0.9 (relating to an odds ratio of 1, 1.1, and 2.45, respectively) and continuous factors a *β* of either 0 (no effect), 0.1 (small effect), or 0.3 (large effect).

All scenarios considered are listed in Appendix B at www.jclinepi.com. In each scenario, we generated 1,000 IPD meta-analysis data sets and then fitted models (1) and (2) to each and recorded $\hat{\beta }$ and its standard error. Each model's performance was then examined by calculating the bias, mean square error (MSE), mean standard error, and coverage for $\hat{\beta }$.

### 4.2 Simulation results

The simulation results for scenarios with five studies and small samples sizes are summarized in Tables 4 and 5, and Appendix C at www.jclinepi.com. The findings were very similar when the number of studies was changed to 10 or when a larger sample size was allowed.

**Fig. 1:**
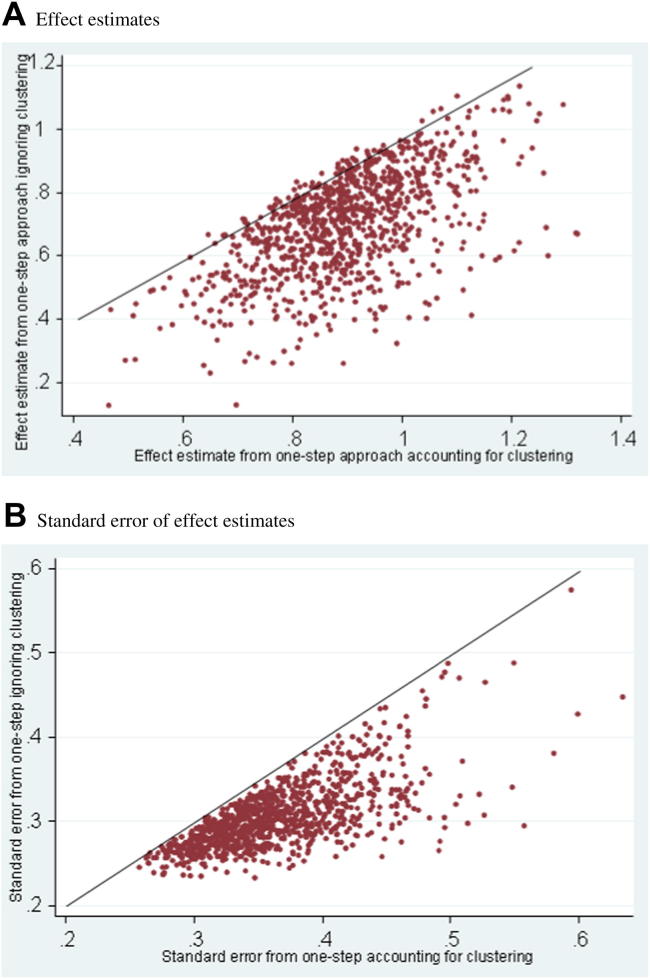
Comparison of the 1,000 simulation results from the one-step accounting clustering vs. the one-step ignoring clustering for scenario 13 with five studies, small study sample sizes, and a binary factor, in which the standard deviation of alpha was 1.5, the true beta was 0.9, and the prevalence was 0.2. (A) Effect estimates. (B) Standard error of effect estimates.

For both binary (Table 4) and continuous factors (Table 5), when there was zero or small variation in baseline risk (*α*_*i*_), the performance of the models was very similar. The bias in $\hat{\beta }$ was close to zero, the MSE was approximately the same, and the coverage was always close to 95%. When the variation in *α*_*i*_ was large (scenarios 13–18 and 22–24), the one-step approach accounting for clustering continues to perform consistently well with suitable bias and coverage. However, the one-step approach ignoring clustering often performs poorly, with downward bias and low coverage especially when the true effect size was large. For example, in scenario 13 (in which the true *β* was 0.9), the one-step model ignoring clustering has a large downward bias of −0.21 and a low coverage of 87.6%, reflecting a small mean standard error (Table 4). This scenario is illustrated in Fig. 1, which shows the one-step approach ignoring clustering produces smaller standard errors in each meta-analysis and generally (though not always) smaller effect estimates than the one-step approach accounting for clustering.

### 4.3 Link to the applied examples of Section 3

When the two-step approach was fitted to the TBI data, step 1 produced separate alpha estimates in each study. The weighted average of these alphas was −2.1, and their between-study standard deviation was 0.20. Thus, the TBI data mirror closely simulation scenario 19 (Table 5), in which alpha was −2.1, the standard deviation of alpha was 0.2, and the true effect was 0.3. In this scenario, there was no difference between models (1) and (2) in terms of bias, MSE, and coverage, and so it is unsurprising that the TBI application shows very similar model (1) and model (2) results.

In contrast to the TBI example, the DVT and smoking applications showed that ignoring clustering produced a substantially smaller odds ratio estimate and a smaller standard error of $\hat{\beta }$ than other methods (Tables 2 and 3). Variability in baseline risk with only a small number of studies is a potential cause of these differences, and in accordance with some of the simulation results in this situation (Fig. 1), ignoring clustering appears to be producing estimates with a downward bias and low coverage in these examples. Other mechanisms may also be causing differences to occur in these examples, beyond those identified by our simulations, such as between-study variation in the proportion of patients who are factor positive [32].

## 5 Discussion

IPD meta-analyses are increasingly used. Riley et al. [1] found 383 IPD meta-analyses published in the medical literature before March 2009, with an average of 49 articles published/year since 2005. In this article, we have examined the impact of ignoring clustering of patients within studies when analyzing IPD of multiple studies with binary outcomes, in which an odds ratio is of interest. In some situations, statistical inferences do not alter whether clustering is accounted for, as seen in the TBI application. However, there are situations when the approaches can differ substantially in their performance, and this can impact on statistical and clinical inferences. This was seen in the DVT and smoking examples and in our simulations with large between-study variability in baseline risk.

There are two key recommendations from our work. The first is that it is inappropriate to simply ignore the clustering of patients within studies and analyze the IPD as if coming from a single study. When there is large variability in baseline risk, the simulations show that this naive approach leads to a downward bias, with small standard errors that produce a low coverage substantially less than 95%; this problem appears to become worse as the true effect size increases. The DVT example shows that ignoring clustering would lead to a potentially important diagnostic marker for DVT being missed, whereas in the smoking example, the effect of nicotine gum on smoking cessation would have been severely underestimated. Other articles in nonmeta-analysis settings have also identified the danger of ignoring clustering, such as in cluster randomized trials [33,34] and multicentre randomized trials [35]. Steyerberg et al. [36] show that in a logistic regression analysis of a clinical trial with multiple strata, the odds ratio of 0.853 when ignoring clustering is reduced to 0.820 when adjusting for strata, an increase of 25% on the logistic scale. Similarly, Hernandez et al. [37] and Turner et al. [38] show that adjustment for prognostic covariates in logistic regression increases power to detect a genuine effect. Statistically speaking, by ignoring clustering, one specifies a marginal model which assumes all studies have the same baseline risk, but by accounting for clustering, one specifies a conditional model that correctly conditions each patient's response on the study there are in. For logistic models, Robinson and Jewell [39] have shown that marginal models give potentially attenuated (biased) effect estimates and have lower power to detect genuine effects than conditional models. For logistic regression, this phenomenon is also known as noncollapsibility of the odds ratio [40] as conditional odds ratios are typically larger than marginal odds ratios after conditioning on important covariates, with the increase becoming higher as the true odds ratio increases and the number of included important covariates increases. Gail et al. [41] showed analytically and through simulation that Cox and exponential regression models for survival data with censoring also produce downwardly biased treatment effect estimates when important covariates are omitted. For linear regression or generalized linear models with a log link (e.g., Poisson regression), the asymptotic bias from omitting covariates is zero, regardless of the true effect size [41]; yet, even for such models, the precision of effect estimates can still be severely affected by ignoring important covariates (clustering) [39]. Statisticians thus may not be surprised by our findings, but we hope our findings raise awareness to the IPD meta-analysis community, many of whom currently ignore clustering [4,18]. We thus recommend that researchers always account for clustering in their IPD meta-analysis and report how they did so in any subsequent publication.

The second important finding is that the one-step model accounting for clustering performs consistently well in all simulations considered, with bias close to zero and suitable coverage. Based on this, we recommend this method to be routinely chosen to analyze IPD with binary outcomes. The two-step method will often give very similar results, as seen in the examples of Section 3. However, the one-step approach models the exact binomial nature of the data directly [16,17], whereas the two-step approach produces log odds ratio estimates in the first step, which are then assumed normally distributed in the second step. This additional normality assumption may be inappropriate when the number of patients in studies is small and/or when the number of events is small. For this reason, the exact one-stage approach of model (1) is generally more suitable for synthesizing two-by-two tables. The Mantel–Haenszel and Peto methods have also been suggested to overcome this issue [42,43], but model (1) can more easily be extended to include multiple factors and continuous variables so is our preferred method. It can also be easily extended to allow between-study heterogeneity in the effect of interest [16]. One could also allow a random-effects distribution on the baseline risk rather than estimating a separate *α*_*i*_ for each study. This requires an additional distributional assumption to be made for *α*_*i*_ s, and for this reason, we prefer model (1) as described previously. A distribution on the baseline risk is perhaps useful if the baseline risk is itself of interest, but in our examples, the focus was only on the effect of the included factor.

Note that it is not possible to predict the direction of bias induced by ignoring clustering in any single example. For example, our simulations with large variability in baseline risk show that ignoring clustering leads to a downward bias *on average*, but Fig. 1 highlights that in a sole application, the actual estimates when ignoring clustering may occasionally be larger than when accounting for clustering. Indeed, the TBI application had a slightly higher odds ratio when ignoring clustering. Our simulations are also limited to particular choices of parameter values and, like all simulation studies, other permutations of values and alternative scenarios are also possible. In particular, between-study variation in prevalence of the binary factor and/or between-study heterogeneity in effect may reveal different findings [32].

None of our binary factor examples or simulations contained studies with zero events in a particular group as this issue has been examined before [22] and been shown to induce bias in the two-step approach as, unlike the one-step approach [16], it requires a continuity correction to be added. Our simulations and examples also did not consider between-study heterogeneity in effects, but our recommendations are likely to generalize to this setting also [17,44]. We also recognize that IPD meta-analyses are not without limitations. Some covariates may not be available for all IPD studies, and IPD may not be available from all studies requested [45]. In this situation, novel methods may be required to synthesize the IPD effectively [10,46,47].

## 6 Conclusion

We have shown that researchers synthesizing IPD from multiple studies should account for the clustering of patients within different studies. Lumping the IPD into a single data set and naively analyzing as if from a single study can produce misleading effects estimates and clinical conclusions, and the correct approach is a one-step or a two-step IPD meta-analysis that correctly accounts for clustering.

## Figures and Tables

**Table e1:** Summary of the IPD available for examining the association between age and 6-month mortality in patients with traumatic brain injury

Study	Number of patients, *n*_*i*_	Mean age, years, (SD)	Age range, years	Mean age/10 years, ${\bar{x}}_{i}$ (SD)	Number of deaths by 6 months	Proportion of dead at 6 months
1	825	32.76 (12.34)	14–77	3.28 (1.23)	199	0.24
2	959	33.29 (14.36)	12–79	3.33 (1.44)	258	0.27
3	466	40.65 (19.85)	16–92	4.07 (1.99)	188	0.40
4	409	32.35 (13.42)	15–79	3.23 (1.34)	94	0.23

*Abbreviations:* IPD, individual participant data; SD, standard deviation.

**Table e2:** Summary of the IPD available for examining the association between a family history of thrombophilia and a confirmed diagnosis of deep vein thrombosis (DVT) in patients with suspected DVT

Study	Number of patients, *n*_*i*_	Proportion with a family history of thrombophilia	Number of true DVT cases	Proportion with true DVT
1	1,756	0.04	411	0.23
2	532	0.26	91	0.17
3	1,075	0.05	190	0.18
4	436	0.19	61	0.13
5	541	0.03	121	0.22
6	259	0.20	35	0.14

*Abbreviation:* IPD, individual participant data.

**Table e3:** Summary of the IPD available for examining the effect of nicotine gum on the odds of smoking cessation

Study	Total number of patients, *n*_*i*_	Nicotine gum group		Control group, number (proportion of total)	Number who stopped smoking	ln odds ratio (SE)
		Number (proportion of total)	Number who stopped smoking			
1	1,286	402 (0.31)	64	884 (0.69)	88	0.538 (0.177)
2	334	270 (0.81)	21	64 (0.19)	1	1.670 (1.033)

*Abbreviations:* IPD, individual participant data; SE, standard error.

**Table e4:** The simulation scenarios for the simulations that were repeated for 5 or 10 studies per meta-analysis and sample sizes of 30–100 or 30–1,000 per study

**Binary factor scenarios**	*α*	*σ*_*α*_	*β*	Prevalence of *x*_*ik*_ = 1
1	−1.27	0	0.90	0.5
2	−1.27	0	0.10	0.5
3	−1.27	0	0.00	0.5
4	−1.27	0.25	0.90	0.5
5	−1.27	0.25	0.10	0.5
6	−1.27	0.25	0.00	0.5
7	−1.27	0	0.90	0.2
8	−1.27	0	0.10	0.2
9	−1.27	0	0.00	0.2
10	−1.27	0.25	0.90	0.2
11	−1.27	0.25	0.10	0.2
12	−1.27	0.25	0.00	0.2
13	−1.27	1.5	0.90	0.2
14	−1.27	1.5	0.10	0.2
15	−1.27	1.5	0.00	0.2
16	−1.27	1.5	0.90	0.5
17	−1.27	1.5	0.10	0.5
18	−1.27	1.5	0.00	0.5

**Continuous factor scenarios**	*α*	*σ*_*α*_	*β*
19	−2.10	0.2	0.3
20	−2.10	0.2	0.1
21	−2.10	0.2	0
22	−2.10	1.5	0.3
23	−2.10	1.5	0.1
24	−2.10	1.5	0

**Table e5:** Simulation results for all the scenarios involving a binary factor with prevalence of 0.5 or 0.2, small study sample sizes between 30 and 100 participants, *m* = 5 studies in the meta-analysis, the true $\hat{\beta }$ was 0, 0.1, or 0.9, and the standard deviation of *α*_*i*_ was 0, 0.25, or 1.5

Scenario	Meta-analysis model	*α* (SD of *α*)	Prevalence	True *β*	Mean $\hat{\beta }$	Bias of $\hat{\beta }$	MSE of $\hat{\beta }$	Coverage (%) of $\hat{\beta }$	Mean SE of $\hat{\beta }$
1	One-step ignoring clustering	−1.27 (0)	0.5	0.9	0.91	0.01	0.03	94.90	0.16
	One-step accounting for clustering	−1.27 (0)	0.5	0.9	0.92	0.02	0.03	94.70	0.16
2	One-step ignoring clustering	−1.27 (0)	0.5	0.10	0.10	0.00	0.00	95.60	0.16
	One-step accounting for clustering	−1.27 (0)	0.5	0.10	0.10	0.00	0.00	95.60	0.16
3	One-step ignoring clustering	−1.27 (0)	0.5	0	0.00	0.00	0.02	94.90	0.16
	One-step accounting for clustering	−1.27 (0)	0.5	0	0.00	0.00	0.02	94.90	0.16
4	One-step ignoring clustering	−1.27 (0.25)	0.5	0.9	0.90	0.00	0.03	95.20	0.17
	One-step accounting for clustering	−1.27 (0.25)	0.5	0.9	0.91	0.01	0.03	95.30	0.18
5	One-step ignoring clustering	−1.27 (0.25)	0.5	0.1	0.09	−0.01	0.04	94.60	0.18
	One-step accounting for clustering	−1.27 (0.25)	0.5	0.1	0.10	0.00	0.04	94.80	0.19
6	One-step ignoring clustering	−1.27 (0.25)	0.5	0	0.01	0.01	0.04	94.70	0.19
	One-step accounting for clustering	−1.27 (0.25)	0.5	0	0.01	0.01	0.04	94.50	0.19
7	One-step ignoring clustering	−1.27 (0)	0.2	0.9	0.90	0.00	0.10	94.90	0.31
	One-step accounting for clustering	−1.27 (0)	0.2	0.9	0.91	0.01	0.10	94.70	0.31
8	One-step ignoring clustering	−1.27 (0)	0.2	0.10	0.10	0.00	0.08	95.30	0.28
	One-step accounting for clustering	−1.27 (0)	0.2	0.10	0.10	0.00	0.09	95.40	0.29
9	One-step ignoring clustering	−1.27 (0)	0.2	0	0.01	0.01	0.08	95.80	0.28
	One-step accounting for clustering	−1.27 (0)	0.2	0	0.01	0.01	0.08	95.80	0.29
10	One-step ignoring clustering	−1.27 (0.25)	0.2	0.9	0.90	0.00	0.09	95.10	0.30
	One-step accounting for clustering	−1.27 (0.25)	0.2	0.9	0.92	0.02	0.10	95.40	0.31
11	One-step ignoring clustering	−1.27 (0.25)	0.2	0.1	0.08	−0.02	0.12	95.50	0.34
	One-step accounting for clustering	−1.27 (0.25)	0.2	0.1	0.09	−0.01	0.12	94.90	0.35
12	One-step ignoring clustering	−1.27 (0.25)	0.2	0	−0.03	−0.03	0.12	95.20	0.35
	One-step accounting for clustering	−1.27 (0.25)	0.2	0	−0.03	−0.03	0.13	95.10	0.35
13	One-step ignoring clustering	−1.27 (1.5)	0.2	0.9	0.69	−0.21	0.15	87.60	0.31
	One-step accounting for clustering	−1.27 (1.5)	0.2	0.9	0.92	0.02	0.14	94.80	0.36
14	One-step ignoring clustering	−1.27 (1.5)	0.2	0.1	0.07	−0.03	0.12	95.70	0.33
	One-step accounting for clustering	−1.27 (1.5)	0.2	0.1	0.10	0.00	0.17	94.20	0.38
15	One-step ignoring clustering	−1.27 (1.5)	0.2	0	−0.02	−0.02	0.22	94.00	0.33
	One-step accounting for clustering	−1.27 (1.5)	0.2	0	0.00	0.00	0.26	94.00	0.38
16	One-step ignoring clustering	−1.27 (1.5)	0.5	0.9	0.70	−0.20	0.04	46.20	0.09
	One-step accounting for clustering	−1.27 (1.5)	0.5	0.9	0.90	0.00	0.05	94.90	0.11
17	One-step ignoring clustering	−1.27 (1.5)	0.5	0.1	0.08	−0.02	0.04	93.90	0.09
	One-step accounting for clustering	−1.27 (1.5)	0.5	0.1	0.10	0.00	0.05	94.80	0.11
18	One-step ignoring clustering	−1.27 (1.5)	0.5	0	0.00	0.00	0.04	94.90	0.09
	One-step accounting for clustering	−1.27 (1.5)	0.5	0	0.00	0.00	0.05	94.70	0.11

*Abbreviations:* SD, standard deviation; MSE, mean standard error; SE, standard error.

**Table e6:** Simulation results for scenarios involving a continuous factor with small study sample sizes between 30 and 100 participants, *m* = 5 studies in the meta-analysis, the true $\hat{\beta }$ was 0, 0.1, or 0.3, and the standard deviation of *α*_*i*_ was 0.2 or 1.5

Scenario	Meta-analysis model	*α* (SD of *α*)	True *β*	Mean $\hat{\beta }$	Bias of $\hat{\beta }$	MSE of $\hat{\beta }$	Coverage (%) of $\hat{\beta }$	Mean SE of $\hat{\beta }$
19	One-step ignoring clustering	−2.1 (0.2)	0.30	0.30	0.00	0.01	96.29	0.09
	One-step accounting for clustering	−2.1 (0.2)	0.30	0.31	0.01	0.01	96.36	0.09
20	One-step ignoring clustering	−2.1 (0.2)	0.10	0.09	−0.01	0.01	96.58	0.11
	One-step accounting for clustering	−2.1 (0.2)	0.10	0.10	0.00	0.01	96.58	0.11
21	One-step ignoring clustering	−2.1 (0.2)	0	0	0	0.02	95.10	0.12
	One-step accounting for clustering	−2.1 (0.2)	0	0	0	0.02	94.90	0.12
22	One-step ignoring clustering	−2.1 (1.5)	0.30	0.23	−0.07	0.01	84.10	0.09
	One-step accounting for clustering	−2.1 (1.5)	0.30	0.31	0.01	0.01	94.80	0.10
23	One-step ignoring clustering	−2.1 (1.5)	0.10	0.08	−0.02	0.01	95.30	0.10
	One-step accounting for clustering	−2.1 (1.5)	0.10	0.10	0.01	0.01	96.10	0.11
24	One-step ignoring clustering	−2.1 (1.5)	0	0.00	0.00	0.01	95.40	0.11
	One-step accounting for clustering	−2.1 (1.5)	0	0.00	0.00	0.02	95.60	0.12

*Abbreviations:* SD, standard deviation; MSE, mean standard error; SE, standard error.

**Table 1:** Traumatic brain injury results for the association between age 10 years and the odds of 6-month mortality, for each of the three IPD models

Methods	$\hat{\beta }$ (SE)	Odds ratio	95% CI for odds ratio	*P*-value
Two-step	0.316 (0.030)	1.372	1.295, 1.454	<0.001
One-step ignoring clustering	0.341 (0.029)	1.407	1.329, 1.488	<0.001
One-step accounting for clustering	0.317 (0.029)	1.373	1.296, 1.455	<0.001

*Abbreviations:* IPD, individual participant data; SE, standard error; CI, confidence interval.

**Table 2:** Results for the effect of a family history of thrombophilia on the odds of truly having deep vein thrombosis, for each of the three IPD models

Methods	$\hat{\beta }$ (SE)	Odds ratio	95% CI for odds ratio	*P*-value
Two-step	0.280 (0.135)	1.323	1.015, 1.725	0.038
One-step ignoring clustering	0.060 (0.128)	1.062	0.825, 1.365	0.642
One-step accounting for clustering	0.263 (0.136)	1.301	0.996, 1.697	0.053

*Abbreviations:* IPD, individual participant data; SE, standard error; CI, confidence interval.

**Table 3:** Results for the effect of nicotine gum on the odds of giving up smoking

Methods	$\hat{\beta }$ (SE)	Odds ratio	95% CI for odds ratio	*P*-value
Two-step	0.570 (0.174)	1.769	1.257, 2.488	0.001
One-step ignoring clustering	0.355 (0.161)	1.400	1.020, 1.916	0.037
One-step accounting for clustering	0.589 (0.170)	1.802	1.290, 2.517	0.001

*Abbreviations:* SE, standard error; CI, confidence interval.

**Table 4:** Simulation results for some of the scenarios involving a binary factor with prevalence of 0.5 or 0.2; small study sample sizes between 30 and 100 participants; *m* = 5 studies in the meta-analysis; the true $\hat{\beta }$ was 0, 0.1, or 0.9; and the standard deviation of *α*_*i*_ was 0, 0.25, or 1.5

Scenarios	Meta-analysis model	*α* (SD of *α*)	Prevalence	True *β*	Mean $\hat{\beta }$	Bias of $\hat{\beta }$	MSE of $\hat{\beta }$	Coverage (%) of $\hat{\beta }$	Mean SE of $\hat{\beta }$
1	One-step ignoring clustering	−1.27 (0)	0.5	0.9	0.91	0.01	0.03	94.90	0.16
	One-step accounting for clustering	−1.27 (0)	0.5	0.9	0.92	0.02	0.03	94.70	0.16
3	One-step ignoring clustering	−1.27 (0)	0.5	0	0.00	0.00	0.02	94.90	0.16
	One-step accounting for clustering	−1.27 (0)	0.5	0	0.00	0.00	0.02	94.90	0.16
13	One-step ignoring clustering	−1.27 (1.5)	0.2	0.9	0.69	−0.21	0.15	87.60	0.31
	One-step accounting for clustering	−1.27 (1.5)	0.2	0.9	0.92	0.02	0.14	94.80	0.36
15	One-step ignoring clustering	−1.27 (1.5)	0.2	0	−0.02	−0.02	0.22	94.00	0.33
	One-step accounting for clustering	−1.27 (1.5)	0.2	0	0.00	0.00	0.26	94.00	0.38
16	One-step ignoring clustering	−1.27 (1.5)	0.5	0.9	0.70	−0.20	0.04	46.20	0.09
	One-step accounting for clustering	−1.27 (1.5)	0.5	0.9	0.90	0.00	0.05	94.90	0.11
18	One-step ignoring clustering	−1.27 (1.5)	0.5	0	0.00	0.00	0.04	94.90	0.09
	One-step accounting for clustering	−1.27 (1.5)	0.5	0	0.00	0.00	0.05	94.70	0.11

*Abbreviations:* SD, standard deviation; MSE, mean square error; SE, standard error.

**Table 5:** Simulation results for scenarios involving a continuous factor with small study sample sizes between 30 and 100 participants; *m* = 5 studies in the meta-analysis; the true $\hat{\beta }$ was 0, 0.1, or 0.3; and the standard deviation of *α*_*i*_ was 0.2 or 1.5

Scenarios	Meta-analysis model	*α* (SD of *α*)	True *β*	Mean $\hat{\beta }$	Bias of $\hat{\beta }$	MSE of $\hat{\beta }$	Coverage (%) of $\hat{\beta }$	Mean SE of $\hat{\beta }$
19	One-step ignoring clustering	−2.1 (0.2)	0.30	0.30	0.00	0.01	96.29	0.09
	One-step accounting for clustering	−2.1 (0.2)	0.30	0.31	0.01	0.01	96.36	0.09
21	One-step ignoring clustering	−2.1 (0.2)	0	0	0	0.02	95.10	0.12
	One-step accounting for clustering	−2.1 (0.2)	0	0	0	0.02	94.90	0.12
22	One-step ignoring clustering	−2.1 (1.5)	0.30	0.23	−0.07	0.01	84.10	0.09
	One-step accounting for clustering	−2.1 (1.5)	0.30	0.31	0.01	0.01	94.80	0.10
24	One-step ignoring clustering	−2.1 (1.5)	0	0.00	0.00	0.01	95.40	0.11
	One-step accounting for clustering	−2.1 (1.5)	0	0.00	0.00	0.02	95.60	0.12

*Abbreviations:* SD, standard deviation; MSE, mean square error; SE, standard error.

## Appendix B Simulation procedure and evaluation

Our simulation procedure can be broken down in six steps as follows:

**Step 1**

We chose the number of studies (*m)* in the meta-analyses, and this was fixed in any simulation. We consider either *m* = 5 or *m* = 10, the typical size of most meta-analyses in our experience.

**Step 2**

We randomly sampled the number of patients in each study from a uniform distribution *n*_*i*_ ∼ *U*(*a*,*b*), with *a* and *b* fixed in any simulation. We used either a small sample size setting using *a* = 30 and *b* = 100 or an enabled larger sample sizes using *a* = 30 and *b* = 1,000.

**Step 3**

*(i) for a binary x*_*ik*_: For each patient in each trial, we randomly sampled a binary factor value, *x*_*ik*_, using a Bernoulli distribution, with *x*_*ik*_ ∼Bernoulli(prevalence). The prevalence denotes the underlying proportion in the study population with *x*_*ik*_ = 1. The prevalence was assumed the same in all studies and fixed in any simulation as either 0.5 or 0.2.

*(ii) for a continuous x*_*ik*_: For each patient in each trial, we randomly sampled a continuous factor value, *x*_*ik*_, using a normal distribution with *x*_*ik*_ ∼ *N*(4, 1.5^2^). The mean and variance were chosen to reflect the distribution of age/10 values in the TBI dataset (Appendix A).

**Step 4**

We randomly sampled the binary outcome *y*_*ik*_ (1, event; 0, alive) for each patient assuming that *y*_*ik*_ ∼Benoulli(*p*_*ik*_) where logit (*p*_*ik*_) = *α*_*i*_ + *βx*_*ik*_. To achieve this, in each simulation, we sampled a value for *α*_*i*_ using $\alpha_{i}∼N(\alpha ,\sigma_{\alpha }^{2})$ and chose a value for *β* (the true effect size, i.e., the log odds ratio). Then

*(i) for a binary factor:* We always chose *α* as −1.27, which is based on the DVT data and relates to a probability of the event of 0.22 for patients with *x*_*ik*_ = 0. Then, *σ*_*α*_ was chosen as 0, 0.25, or 1.5, and *β* was 0, 0.1, or 0.9 (relating to an odds ratio of 1, 1.1, and 2.45, respectively). The chosen $\sigma_{\alpha }^{2}$ values covered zero, small, or large between-study variability in baseline risk, and the chosen *β* values covered a zero, small, or large prognostic effect. When $\sigma_{\alpha }^{2}$ was 1.5, the 95% range for the baseline log odds of the event across studies is between −1.27 ± (1.96*1.5), which translates to a range in baseline event probability from 0.01 to 0.85. Clearly, this is extreme but is deliberately chosen to view the impact in such a setting. It may also occur when case–control studies are synthesized as the researcher then samples based on event status and thus influences the proportion of patients with events in each group (and thus influences their *α*_*i*_).

*(ii) for a continuous factor:* We always chose *α* as −2.10, which is based on the TBI data and relates to a probability of the event at age zero of 0.11 for patients with *x*_*ik*_ = 0. Either small (0.2) or large (1.5) variability in *α* was chosen, and a one-unit increase in *x*_*ik*_ (e.g., an increase in 10 years when *x*_*ik*_ relates to age/10) increased the log odds by 0 (no effect), 0.1 (small effect), or 0.3 (large effect).

**Step 5**

We repeated steps 1–4 until 1,000 IPD meta-analysis data sets had been generated, keeping the chosen range of sample sizes, number of studies, and parameter values as before in each step.

**Step 6**

To each of the 1,000 IPD meta-analysis data sets generated from steps 1 to 5, we fitted each of models (1) and (2) and recorded $\hat{\beta }$ and its standard error on each occasion.

**Simulation scenarios**

Steps 1–6 were repeated for a range of different simulation scenarios (see table below), according to different permutations and choices of *m*, *a*, *b*, $\sigma_{\alpha }^{2}$, β, continuous, or binary *x*_*ik*_, and if binary, the prevalence of *x*_*ik*_ = 1. For example, for the evaluation of a binary factor, in total 72 different simulation settings were evaluated for each combination of 5 or 10 studies, with small (30–100) or large (30–1,000) sample sizes, and the choice of $\sigma_{\alpha }^{2}$, *β*, and prevalence. Each simulation scenario took between 4 and 14 hours to run, with the longer times required for 10 studies and the larger sample sizes.

**Evaluating model performance**

For each simulation scenario, 1,000 values for $\hat{\beta }$ and its standard error were available for each model after step 6, and the corresponding 1,000 confidence intervals were calculated using $\hat{\beta }\pm 1.96\sqrt{\mathrm{var}\left(\hat{\beta }\right)}$. Assessment of each model’s performance was then examined by calculating the bias, MSE, mean standard error, and coverage for $\hat{\beta }$. The estimated coverage was the proportion of the 1,000 simulations in which the 95% confidence interval contained the true *β*. Note that, because of sampling variability from using “only” 1,000 simulations, coverage can deviate from 95% by chance, even when the true coverage is 95%. Assuming the coverage truly was 95%, we expected to observe a coverage proportion between 0.95 ± (1.96 × 0.00689) = [0.936, 0.964] in each simulation, where 0.00689 is the standard error of an estimated coverage of 0.95 from 1,000 simulations. Thus, coverage values outside the range of 93.6–96.4% were considered as indicative of poor parameter estimation for *β*.
